# The Epl1 and Sm1 proteins from *Trichoderma atroviride* and *Trichoderma virens* differentially modulate systemic disease resistance against different life style pathogens in *Solanum lycopersicum*

**DOI:** 10.3389/fpls.2015.00077

**Published:** 2015-02-23

**Authors:** Miguel A. Salas-Marina, María I. Isordia-Jasso, María A. Islas-Osuna, Pablo Delgado-Sánchez, Juan F. Jiménez-Bremont, Margarita Rodríguez-Kessler, María T. Rosales-Saavedra, Alfredo Herrera-Estrella, Sergio Casas-Flores

**Affiliations:** ^1^Laboratorio de Genómica Funcional y Comparativa, División de Biología Molecular, Instituto Potosino de Investigación Científica y TecnológicaSan Luis Potosí, Mexico; ^2^Laboratorio de Genética y Biología Molecular, Centro de Investigación en Alimentación y Desarrollo, Dirección Tecnología de Alimentos de Origen VegetalHermosillo, Mexico; ^3^Laboratorio Nacional de Genómica para la BiodiversidadIrapuato, Mexico

**Keywords:** Trichoderma, tomato, Sm1/Epl1, biotrophic phytopathogen, necrotrophic phytopathogen, systemic acquired resistance, induced systemic resistance

## Abstract

Fungi belonging to the genus *Trichoderma*, commonly found in soil or colonizing plant roots, exert beneficial effects on plants, including the promotion of growth and the induction of resistance to disease. *T. virens and T. atroviride* secrete the proteins Sm1 and Epl1, respectively, which elicit local and systemic disease resistance in plants. In this work, we show that these fungi promote growth in tomato (*Solanum lycopersicum*) plants. *T. virens* was more effective than *T. atroviride* in promoting biomass gain, and both fungi were capable of inducing systemic protection in tomato against *Alternaria solani*, *Botrytis cinerea*, and *Pseudomonas syringae* pv. *tomato* (*Pst* DC3000). Deletion (KO) of *epl1* in *T. atroviride* resulted in diminished systemic protection against *A. solani* and *B. cinerea*, whereas the *T. virens sm1* KO strain was less effective in protecting tomato against *Pst* DC3000 and *B. cinerea*. Importantly, overexpression (OE) of *epl1* and *sm1* led to an increase in disease resistance against all tested pathogens. Although the *Trichoderma* WT strains induced both systemic acquired resistance (SAR)- and induced systemic resistance (ISR)-related genes in tomato, inoculation of plants with OE and KO strains revealed that Epl1 and Sm1 play a minor role in the induction of these genes. However, we found that Epl1 and Sm1 induce the expression of a peroxidase and an α-dioxygenase encoding genes, respectively, which could be important for tomato protection by *Trichoderma* spp. Altogether, these observations indicate that colonization by beneficial and/or infection by pathogenic microorganisms dictates many of the outcomes in plants, which are more complex than previously thought.

## Introduction

To counteract pathogens, plants have developed several layers of immune responses, including detection of pathogen-associated molecular patterns (PAMPs) that induce PAMP-triggered immunity (PTI). In turn, pathogens have evolved effector molecules to suppress PTI to survive and spread into the host. Facing this challenge, plants have developed resistance proteins (R) that recognize pathogen effectors and promote effector-triggered immunity (ETI) (Chisholm et al., 2006; Katiyar-Agarwal and Jin, 2010). Furthermore, plants have developed the ability to enhance their basal resistance after detection of a pathogen. This response includes systemic acquired resistance (SAR) and induced systemic resistance (ISR) that are phenotypically similar but significantly different at the genetic and biochemical levels. SAR is associated with the accumulation of salicylic acid (SA) (Durrant and Dong, 2004; Glazebrook, 2005), whereas ISR is a response to the accumulation of jasmonic acid (JA) and ethylene (ET) (Ton et al., 2002). The SA-related defense response is triggered against biotrophic and hemibiotrophic pathogens, such as *Pseudomonas syringae* pv. *tomato* DC3000 (*Pst* DC3000), which feeds on living host tissue, and is accompanied by the accumulation of pathogenesis-related proteins (PR) (Van Loon and Van Strien, 1999; Durrant and Dong, 2004; Glazebrook, 2005). The JA/ET-related defense response is boosted by necrotrophic microorganisms such as *Botrytis cinerea*, which kills the host tissue at early stages of the invasion (Ton et al., 2002); genes related to ISR such as *PDF1.2* and *LOX-2* are induced upon infection by these type of pathogens. Beneficial soilborne microorganisms such as rhizobacteria, mycorrhizae, and non-pathogenic fungi can also induce plant systemic resistance against a broad spectrum of microbial pathogens, where JA/ET have shown to be the major players (Van Wees et al., 2008).

Fungi belonging to the *Trichoderma* genus are free-living organisms commonly found in soil, decaying wood, or colonizing the plant root surface (Brotman et al., 2010; Druzhinina et al., 2011; Hermosa et al., 2012). Root colonization by *Trichoderma* provides significant beneficial effects to plants, including changes in root architecture, growth enhancement, and an increase in productivity (Baker et al., 1984; Chang et al., 1986; Harman, 2000). Activation of systemic resistance against phytopathogens by *Trichoderma* has been reported for both monocot (Djonovic et al., 2007; Shoresh and Harman, 2008) and dicot plants (Yedidia et al., 2003; Shoresh et al., 2005; Viterbo et al., 2005; Djonovic et al., 2006). This response was described as the closest analog of the ISR activated by rhizobacteria, and is associated with the accumulation of JA/ET, and the transcription of ISR-related genes (Bakker et al., 2003; Van Loon, 2007). Contrasting data have shown that *T. longibrachiatum* induces the expression of PR genes (Martinez et al., 2001). Even though SA and JA/ET pathways are mutually antagonistic, evidences of synergistic interactions have been demonstrated (Mur et al., 2006). Recently, simultaneous induction of genetic markers from the SAR and ISR pathways in *Arabidopsis thaliana* by *Trichoderma* has been reported (Contreras-Cornejo et al., 2011; Salas-Marina et al., 2011). Additional studies in more plants have shown simultaneous induction of the SA and JA/ET pathways by other *Trichoderma* species (Mathys et al., 2012; Perazzolli et al., 2012; Cai et al., 2013; Olmedo-Monfil and Casas-Flores, 2014). Recently, it was shown that intact JA, ET and abscisic acid (ABA) signaling pathways are required for functional induction of systemic resistance in tomato plants against *B. cinerea* (Martínez-Medina et al., 2013).

At the beginning of plant root colonization, *Trichoderma* is probably recognized initially as foreign, through its microbial-associated molecular patterns (MAMPs), which lead to the induction of plant systemic resistance. Indeed, growing evidence suggests that during the plant-*Trichoderma* interaction a molecular dialog takes place, likely mediated by molecules produced by both the plant and *Trichoderma* (Pozo et al., 2005). A number of MAMPs have been characterized in these fungi, including oligosaccharides, low molecular weight compounds, proteins with enzymatic activity, swollenins, peptaibols, and cerato-platanins (Bailey et al., 1991; Baker et al., 1997; Djonovic et al., 2006; Viterbo et al., 2007; Brotman et al., 2008). Among these molecules, the Sm1 (small protein -1) from *T. virens* and its homologous Epl1 (eliciting plant response-like) from *T. atroviride* are of particular interest. They are proteinaceous non-enzymatic elicitors of plant disease resistance that belong to the cerato-platanin family, and are abundantly expressed in the absence of plants (Djonovic et al., 2006, 2007; Seidl et al., 2006). However, their expression increases in the presence of plants, and their products are secreted at the early stages of their interaction, suggesting a signaling role of such proteins during these relationships (Djonovic et al., 2006; Vargas et al., 2008). In support of this hypothesis, purified Sm1 protein was found to induce locally and systemically the cotton defense response, whereas the expression of *sm1* was shown to be essential for the induction of systemic resistance in maize against the foliar pathogen *Colletotrichum graminicola*. The proteins Sm1 and Epl1 are produced mainly as monomer and a dimer, respectively, in the presence of maize plants, but the monomeric form is responsible for the induction of systemic resistance (Vargas et al., 2008). The protective activity of Sm1 has been associated with the induction of the JA pathway and green leaf volatile-biosynthetic genes (Djonovic et al., 2006).

The present study was conducted to investigate the effects of *T. atroviride* and *T. virens* on the growth and the induction of systemic disease resistance against different foliar pathogens in tomato plants. Furthermore, we studied the effect of the Sm1 and Epl1 elicitors on disease resistance in the same plants. For this purpose, we generated *sm1* and *epl1* overexpression (OE) and knockout (KO) strains of the two fungi. Then, we determined the induction of systemic disease resistance against different pathogens and measured the expression of tomato defense-related genes after inoculation with the different *Trichoderma* strains. These data enabled us to establish new insights into systemic disease resistance induced by *Trichoderma* spp. and the role of the Sm1 and Epl1 elicitors in this process.

## Materials and methods

### Microorganisms and growth conditions

*T. virens* Gv29-8 (Baek and Kenerley, 1998) and *T. atroviride* IMI 206040 were used throughout this study. *B. cinerea* and *A. solani* strains were isolated from a tomato plant in San Luis Potosi, Mexico, and identified by PCR amplification of 18S rDNA using the oligonucleotides ITS1 and ITS4 (White et al., 1990). Fungal strains were routinely maintained on potato dextrose agar (PDA) (Difco, Franklin Lakes, NJ, USA), and hygromycin was added at 200 μg/ml when necessary. *Pst* DC3000 (Cuppels, 1986) was routinely grown on King's B medium (King et al., 1954). *Escherichia coli* Top 10 F' was routinely grown in Luria-Bertani (LB) broth or on LB agar plates. Carbenicillin (100 μg/ml) was added to LB when necessary. *E. coli* Top 10 F' was used for DNA manipulations (Sambrook and Russell, 2001).

### Plant-growth promotion assay

Tomato seeds were plated on 0.3× MS (Murashige and Skoog, 1962) and, 4 days after germination, seedlings were transplanted to flowerpots containing peat moss as substrate (LAMBERT™), and roots were inoculated with 20μl of 1 × 10^6^ spores ml^−1^ of *T. atroviride* or *T. virens*. One day post-inoculation (dpi), flowerpots were irrigated with liquid MS (0.3×) to allow the fungus to colonize the rhizosphere. Six dpi, plants were supplied with nutrient solution HUMIFERT (Cosmocel, Monterrey, NL, Mexico) (0.3%). Twenty-one dpi with *Trichoderma*, control and treated plants were carefully removed from containers and roots were washed in sterile distilled water. Plant length was measured with a ruler and fresh weight was determined on an analytical scale. Then, plants were air-dried at 70°C for 72 h to further measure the dry weight on an analytical scale. Each treatment consisted of 15 plants, and the experiment was repeated three times.

### Protection assay against fungal and bacterial phytopathogens induced by *T. atroviride* and *T. virens* strains

Phytopathogenic fungi were grown for 7 days on PDA at 28°C with a 12 h photoperiod. Conidia were harvested and suspended in sterile distilled water. Conidia were counted by using a hematocytometer and the spore suspension was adjusted to 1 × 10^6^ and 1 × 10^5^ conidia ml^−1^ for *B. cinerea* and for *A. solani*, respectively. *Pst* DC3000 was grown in King's B medium at 200 rpm for 48 h at 28°C and the suspension was adjusted to OD = 0.2. Break-Thru (Goldsmidt Chemical Corporation) was added to a final concentration of 0.1% as surfactant agent. Tomato plants used for protection assays were grown as described for plant growth promotion trials. Twenty-one dpi of tomato with the different *Trichoderma* strains, the plants were inoculated with *B. cinerea*, *A. solani*, or *P*. *syringae*. For each treatment, we used 8 plants. Three leaves from each plant were inoculated with 10 μl of the pathogen suspension on the adaxial side and on the mid vein of the leaf. Inoculated plants were placed in the greenhouse under controlled conditions and irrigated daily. Eight dpi with the pathogen, leaf damage area was measured with a transparent grid (4 mm^2^ grid squares). Percentage of leaves damage area was calculated obtaining the total leaf area and the total damaged leaf area, the ratio between these values gave the percentage of damaged area. Each experiment was repeated three times. Experimental data were subjected to analysis of variance, setting significance at *P*-values < 0.0001, LSD range test < 0.05.

### Generation of *sm1* and *epl1* overexpression and deletion constructs

Total DNA from *T. virens* and *T. atroviride* was extracted as described by Raeder and Broda (1989). The *sm1* and *epl1* gene deletion constructs were generated through the double-joint PCR tool (Yu et al., 2004). In a first round of PCR, ~1.5 kb of each of 5′- and 3′-flanking regions for the *sm1* and *epl1* open reading frames were amplified from genomic DNA from *T. virens* and *T. atroviride*, respectively, using the primers enlisted in Table 1. The 1.4-kb *hph* cassette was PCR-amplified from the plasmid pCB1004 (Carroll et al., 1994) using primers hph-f and hph-r (Table 1). The *sm1* and *epl1* 5′ and 3′ open reading frame flanking regions were mixed with the *hph* amplicon in a 1:1:3 molar ratios and the second round of PCR was performed as described elsewhere (Yu et al., 2004). For generating *sm1* and *epl1* overexpression strains, the primers listed in Table 1, were used to amplify the *sm1* and *epl1* genes (Table 1). The forward and reverse primers included the *Xba* I and *Nsi* I restriction sites, respectively. The *sm1* and *epl1* amplicons were double digested with *Xba* I and *Nsi* I and cloned into the pGFP-Hyg vector in their corresponding restriction sites under regulation of the pyruvate kinase gene (*pki*) promoter from *T. reesei* (Zeilinger et al., 1999; Casas-Flores et al., 2006). PCR amplification of *sm1*/*epl1* was carried out under the following conditions (°C/t): 1 cycle 94/5 min, 25 cycles 94/30 s, 60/30 s, 72/30 s, and one final cycle of 72/10 min. The PCR products were verified by sequencing.

**Table 1:** **Oligonucleotides used in this work**.

**Primer name**	**Sequence (5′-3′)^**a**^**	**Gene amplified**	**GenBank accession number**
Tasm1OE-f	GCTCTAGAATGCAACTGTCCAACATCTTCACTC	*epl1*	AJ901879.1
Tasm1OE-r	CCAATGCATTTAGAGACCGCAGTTCTTAACAGG	*epl1*	AJ901879.1
Tvsm1OE-f	GCTCTAGAATGCAGTTCTCCAGCCTCTTCAAG	*sm1*	DQ121133.1
Tvsm1OE-r	CCAATGCATTTAGAGGCCGCAGTTGCTCACAGC	*sm1*	DQ121133.1
Tasm1KO5′-f	CGGGATCCGCACTGGGTAGATGCTGGTCTG	*epl1*	AJ901879.1
Tasm1KO5′-r	CTCCTTCAATATCAGTTAACGTCGATCCTGAGTAGTGAAGCGAATGTGCTG	*epl1*	AJ901879.1
Tasm1KO3′-f	CAGCACTCGTCCGAGGGCAAAGGAATAGCGGAGCAATGTAAGCAGATCGAC	*epl1*	AJ901879.1
Tasm1KO3′-r	CCGCTCGAGCCTTACTGCAAAGGGTCTGGATGC	*epl1*	AJ901879.1
Tvsm1KO5′-f	GCTCTAGAACAATGCCGGTAGTACACCGTTCG	*sm1*	DQ121133.1
Tvsm1KO5′-r	CTCCTTCAATATCAGTTAACGTCGATCGGGTACAGCAAACTGACTCGTCAC	*sm1*	DQ121133.1
Tvsm1KO3′-f	CAGCACTCGTCCGAGGGCAAAGGAATAGCGACCAGTAAACCGCCATTCATCG	*sm1*	DQ121133.1
Tvsm1KO3′-r	CCGCTCGAGGGACTTGTCGAATTTCCCATCTCG	*sm1*	DQ121133.1
*Ta-TEF-1-Fw*	AGGCCGAGCGTGAGCGTGGTAT	*ef-1 alpha*	ID 146236
*Ta-TEF-1-Rv*	ATGGGGACGAAGGCAACGGTCTT	*ef-1 alpha*	ID 146236
hph-f	GATCGACGTTAACTGATATTGAAGGAG	*hph*	X03615.1
hph-r	CTATTCCTTTGCCCTCGGACGAGTGCTG	*hph*	X03615.1
GLUA-F	GTGAAGCTGGTTTGGGAAATG	*SlGLUA*	M80604.1
GLUA-R	TTGCCAATCAACGTCATGTCTAC	*SlGLUA*	M80604.1
PR-5-F	GGTGCCAGACTGGTGATTGTG	*SlPR-5*	AY093595.1
PR-5-R	TTGGTGGTTTACCCCATCCTT	*SlPR-5*	AY093595.1
DOX1-F	TCACACCATAGATTGGACTGTTCA	*Slα-DOX1*	AY344539.1
DOX1-R	GGCACGCATTCCTGCAA	*Slα-DOX1*	AY344539.1
CHI9-F	AACGCGGGAATTGTTCGA	*SlCHI9*	Z15140.1
CHI9-R	GCAGGACATGCGTCATTGTT	*SlCHI9*	Z15140.1
TLRP-F	TGCGGTGAAATTGGATAACG	*SlTLRP*	X77373.1
TLRP-R	GCCATAGCCCTTGCCATAATAA	*SlTLRP*	X77373.1
CEVI16-F	AACGGAGATGGCTCGAAGCGTG	*SlCEVI16*	X94943.1
CEVI16-R	CATCGGTCCACAATATCTGGTCTG	*SlCEVI16*	X94943.1
ACT-f	GCTGCAGGTATCCACGAGACTACC	*SlTOM51*	U60481.1
ACT-r	GAT TTC CTT GCT CAT ACG GTC AGC	*SlTOM51*	U60481.1

*^*a*^ Restriction sites are indicated as underlined letters*.

### Genetic transformation of *T. atroviride* and *T. virens* protoplasts

Protoplasts of *T. virens* and *T. atroviride* were transformed with overexpression and deletion constructs, as described elsewhere (Baek and Kenerley, 1998). Stable transformants were selected by three consecutive transfers of a single colony to PDA medium plus 200 μg/ml hygromycin. To estimate the *sm1* gene replacement and the copy numbers of the overexpression strains, the 2^−ΔΔCt^ method was assessed (Livak and Schmittgen, 2001). Genomic DNA extracted from wild-type strains was used as calibrator, whereas *ef-1 alpha* gene served as housekeeping in all experiments. After validation of the method, results were expressed in N-fold changes in the target gene copies normalized to *ef-1 alpha* relative to the copy number of the target gene in *T. virens* and *T. atroviride* using the equation 2^−ΔΔCt^ (Livak and Schmittgen, 2001). Two experiments were carried out for each sample in triplicate and the Ct was recorded. Real-time PCR was performed using SYBR Green Fast SYBR technology on the 7500 FAST Real-Time PCR System (Applied Biosystems), following the default PCR program.

### RT-qPCR analysis of the *sm1* and *epl1* overexpression and deletion strains

The different *Trichoderma* strains were grown on PDA plates overlaid with a sterile cellophane sheet, incubated for 3 days at 28°C, and mycelia were harvested for total RNA extraction using TRIzol Reagent (Invitrogen), as described by the manufacturer. Briefly, 2 μg of total RNA was treated with rDNase I (Ambion) and reverse-transcribed to cDNA with SuperScript II Reverse Transcriptase (Invitrogen) using oligo-dT primer. The synthesized cDNAs concentration were checked in a Nanodrop spectrophometer (Thermo Scientific) and used as template for real time RT-PCR.

### Expression analysis of tomato defense related genes

Fourteen-day-old plants grown in Petri dishes containing MS medium were inoculated with 10 μl of 1 × 10^6^ conidia ml^−1^ of *T. virens* WT, TvOE2.2, TvKO2, *T*. *atroviride* WT, TaOE2.1, and TaKO9 strains. At 72 h post *Trichoderma* inoculation, tomato roots and leaves were separated and frozen immediately in liquid nitrogen. Total RNA was extracted by using the Concert RNA extraction reagent (Invitrogen) as recommended by the manufacturer. Total RNA (2 μg) was DNase-treated using rDNase I (Ambion) and reverse-transcribed with SuperScript II Reverse Transcriptase (Invitrogen), and used for real-time RT-PCR as described before. Six tomato genes related to different plant defense pathways were selected from a subtractive library (*SlCHI9*, *SlTLRP*, *Slα-DOX1*, *SlPR-5*, *SlGLUA*) (this work) and two from the GenBank database (*SlCEVI16, SlTOM51*) (Table 1). The subtraction was performed using total RNA from TaOE2.1-treated tomato plants against TaKO9-treated tomato plants. BLAST searches against GeneBank of selected sequences from the subtractive library were performed. The SAR-related genes were: acidic isoform class II β-1, 3-glucanase (*SlGLUA*), osmotin-like (*SlPR-5*). The ISR-related genes were: Class I basic endochitinase (*SlCHI9*), secreted peroxidase (*SlCEVI16*), α–dioxygenase (*Sl*α*-DOX1*), tomato cell wall protein (*SlTLRP*) (related to induced systemic resistance and oxidative burst). Actin gene, *SlTOM51*, was selected as the housekeeping gene (Table 1). Seven primer pairs were designed, using the Primer Express 3 Software (Applied Biosystems) (Table 1).

## Results

### *T. virens* and *T. atroviride* promote growth in tomato (*solanum lycopersicum*)

**Figure 1:**
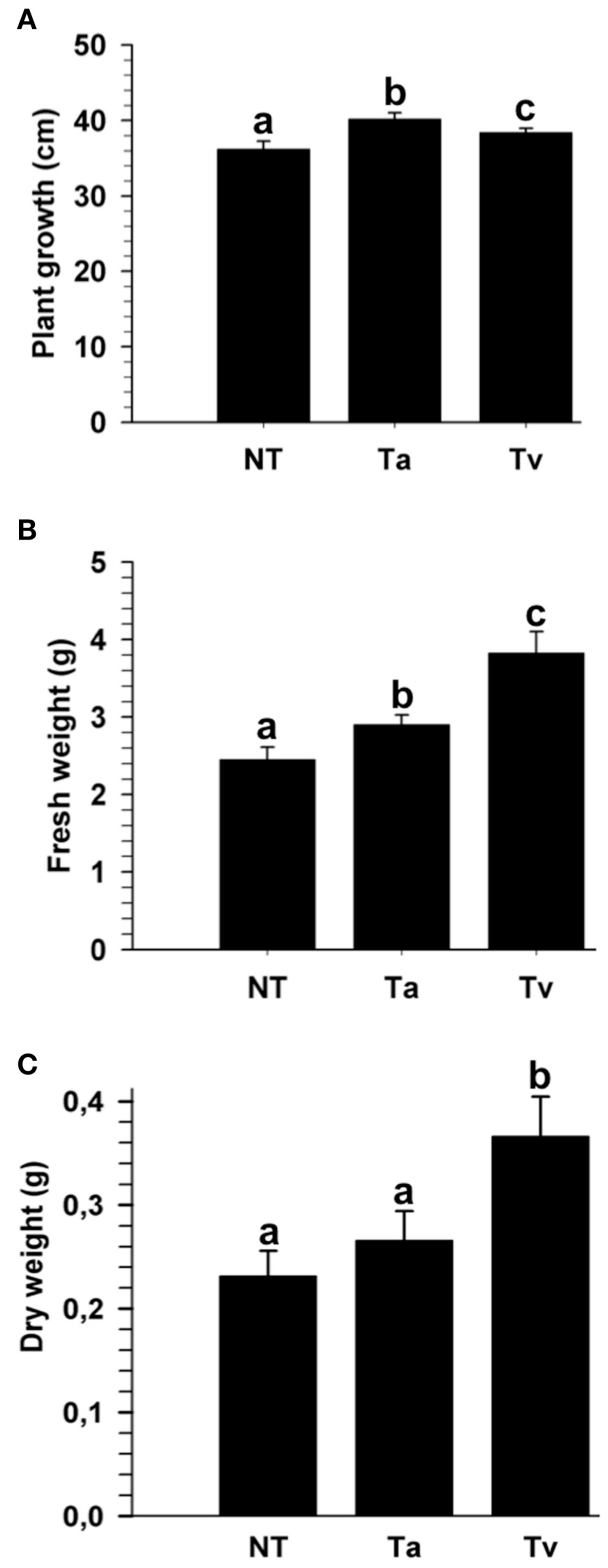
***T. atroviride* and *T. virens* wild-type strains promote growth of tomato plants**. Twenty-one dpi with *T. atroviride* (Ta) or *T. virens* (Tv), the entire plant length was measured **(A)**; and fresh weight **(B)** and dry weight were determined **(C)**. Non-*Trichoderma* inoculated (NT) results are representative of three independent experiments. Letter indicates statistically significant differences (analysis of variance, *P* < 0.0001, LSD range test < 0.05).

Beneficial effects of several *Trichoderma* strains, including *T. atroviride* (IMI 206040) and *T*. *virens* (TvG29-8), on plants have been reported; however, to the best of our knowledge, these strains have not been tested in tomato (*Solanum lycopersicum*). To evaluate the effect of *T. atroviride* and *T. virens* on the growth of tomato plants, 4 days old tomato seedlings were either root treated or not with a conidial suspension of one of the two fungi. After twenty-one dpi with *Trichoderma*, the plant growth and fresh and dry weights were determined. Seedlings treated with either *Trichoderma* species were higher than mock plants (Figure 1 and Figure S1). However, seedlings treated with *T. virens* showed a marked increase in fresh and dry weight compared to plants inoculated with *T*. *atroviride* (Figure 1 and Figure S1).

### Generation of *epl1* and *sm1* deletion and overexpression strains

To determine if the Epl1 and Sm1 proteins play a role in the induction of systemic disease resistance in tomato, e*pl1*- and *sm1*-deletion (KO) and overexpression strains (OE) were generated by gene replacement and by transformation with constructs bearing the *sm1* or *epl1* genes under the control of the piruvate kinase (*pki*) constitutive promoter from *T. reesei* (Zeilinger et al., 1999). Two KO candidate strains for *T. atroviride* (TaKO9 and TaKO11) and two for *T. virens* (TvKO2 and TVKO6) were selected, whereas three *T*. *atroviride* OEs transformants (TaOE1.1, TaOE2.1, and TaOE3.1) and three for *T*. *virens* (TvOE2.1, TvOE2.1, and TvOE6.2) were selected. The *T. atroviride* and *T. virens* KO, OE, and wild-type strains were grown on PDA plates for 10 days, and their phenotypes were inspected visually daily. There were no phenotypic differences on growth rate, colony appearance, or conidiation when compared with their respective wild-type strains (data not shown). Deletion and copy number of *epl1* and *sm1* on the *T. atroviride* and *T. virens* genomes of the transformants and wild-type strains were analyzed with qPCR (quantitative polymerase chain reaction) (Table 2). As expected *T. virens* and *T. atroviride* KO strains lacked *sm1* or *epl1*, whereas the TvOE2.2, TvOE2.1, and TvOE6.2 strains showed three, four, and seven copies of *sm1*, respectively, whilst the TaOE1.1, TaOE3.1, and TaOE2.1 strains showed three, four, and five copies of *epl1* (Table 2).

**Table 2:** ***epl1* and *sm1* copy number and expression levels in the *T. atroviride* and *T. virens* OE, KO and wild type strains, calculated with the 2^-ΔΔCt^ method**.

**Strain**	***epl1*/*sm1* Copy number**	***epl1* relative expression**	***sm1* relative expression**
*T. atroviride* WT	1	1.0	
TaOE1.1	3	14.2	
TaOE2.1	5	8.5	
TaOE3.1	4	4.9	
TaKO9	0	0.0	
TaKO11	0	0.0	
*T. virens* WT	1		1.0
TvOE2.1	4		4.6
TvOE2.2	3		8.1
TvOE6.2	7		0.5
TvKO2	0		0.0
TvKO6	0		0.0

### Gene expression analysis of *epl1* and *sm1* in the OE and KO strains

To test if the *sm1* and *epl1* copy number of the OE strains correlates with the transcription levels of *sm1* and *epl1*, total RNA from the different strains was analyzed by quantitative reverse transcriptase PCR (RT-qPCR). The TaOE1.1, TaOE2.1, and TaOE3.1 strains showed 14.2-, 8.5-, and 4.9-fold (Table 2) transcript levels, whereas the TvOE2.1, TvOE2.2 and TvOE6.2 strains showed 4.6-, 8.1-, and 0.5-fold transcript levels (Table 2). Interestingly, with more than three copies of genes *epl1* or *sm1*, transcript levels of these genes were lower than with three copies (Table 2). The KO strains showed no *epl1* or *sm1* transcript in the corresponding strains (Table 2).

### Sm1 and epl1 differentially modulate the induction of systemic disease resistance against different pathogens in tomato plants

**Figure 2:**
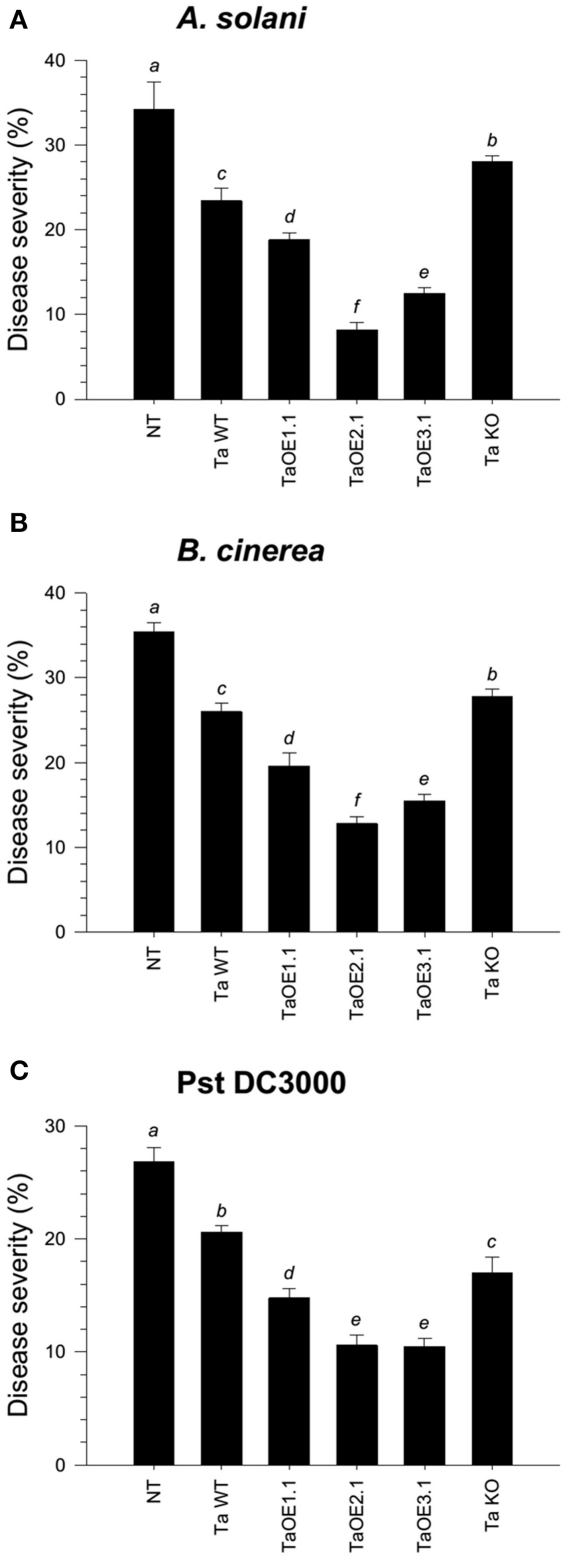
**Epl1 plays a major role in the induction of disease resistance against necrotrophic, but a minor role against hemibiotrophic phytopathogens in tomato**. Twenty-one dpi of tomato with *T. atroviride* WT, TaOE1.1, TaOE2.1, TaOE3.1 and TaKO9 strains, tomato leaves were inoculated with *B. cinerea* **(A)**, *A. solani* **(B)**, and *Pst* DC3000 **(C)**. Non-*Trichoderma* inoculated (NT) foliar damage was evaluated 8 dpi with the phytopathogen, taking the damaged area of three inoculated leaves from a total of eight plants. Each bar represents an average of three independent experiments given as arbitrary units. Letter indicates statistically significant differences (analysis of variance, *P* < 0.0001, LSD range test < 0.05).

**Figure 3:**
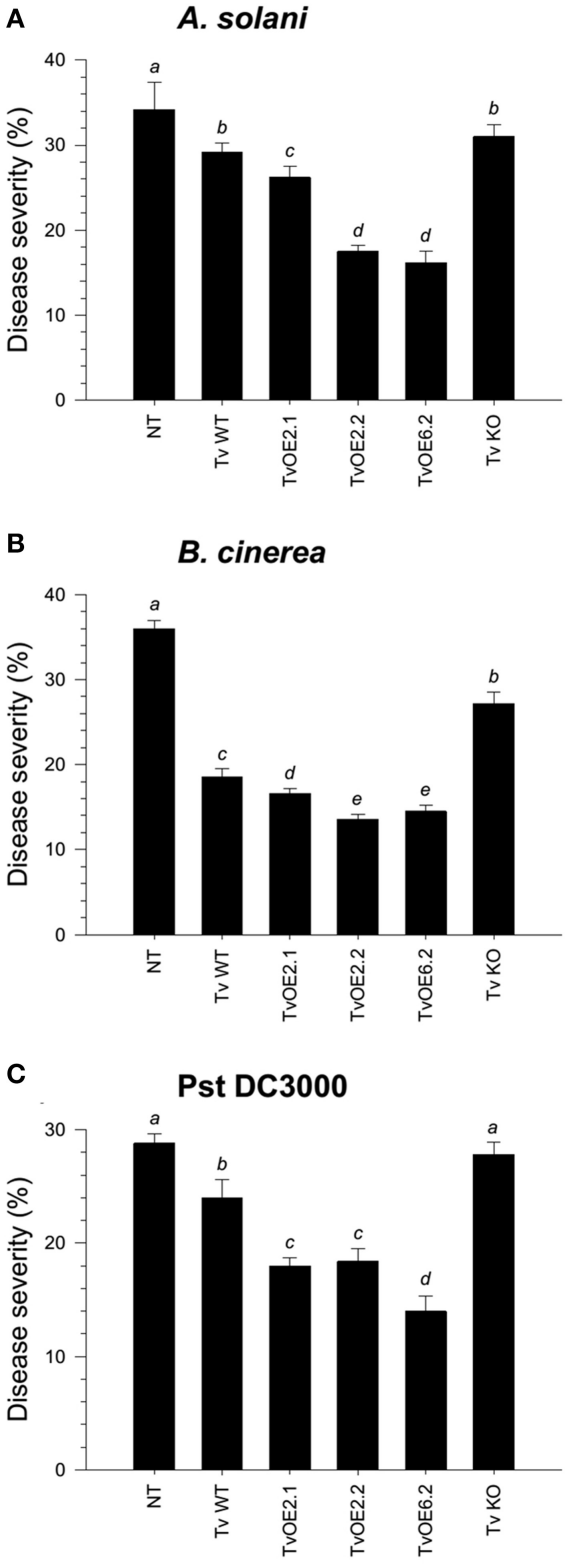
**Sm1 plays a minor role in the induction of disease resistance against necrotrophic, but a major role against hemibiotrophic phytopathogens in tomato**. Twenty-one dpi of tomato with *T. virens* WT, TvOE2.1, TvOE2.2, TvOE6.2 and TvKO2 strains, tomato leaves were inoculated with *B. cinerea* **(A)**, *A. solani* **(B)**, and *Pst* DC3000 **(C)**. Non-*Trichoderma* inoculated (NT) foliar damage was evaluated 8 dpi with the phytopathogen, taking the damaged area of three inoculated leaves from a total of eight plants. Each bar represents an average of three independent experiments given as arbitrary units. Letter indicates statistically significant differences (analysis of variance, *P* < 0.0001, LSD range test < 0.05).

To assess the ability of the different *Trichoderma* strains to systemically protect tomato seedlings against three different foliar pathogens, roots of four-day-old seedlings were inoculated with the whole set of strains. Tomato leaves after twenty-one dpi with *Trichoderma* were infected with a conidial suspension of *Alternaria solani* or *Botrytis cinerea*, or with a bacterial suspension of *Pseudomonas syringae* pv. *tomato* (*Pst* DC3000). Disease lesions on tomato leaves were evaluated eight dpi and the mean of percentage of leaf area damaged was then calculated. The mocked seedlings, inoculated with *A. solani*, showed 34.2% of leaf damage, whereas the *T. atroviride* and *T. virens* wild-type strains-treated seedlings, infected with *A. solani*, showed 23.4% (Figure 2A and Figure S2A) and 29.2% (Figure 3A and Figure S3A) of foliar damage, respectively. The TaOE2.1 treated seedlings infected with *A. solani* showed only 8.2% of foliar damage followed by TaOE3.1- (12.5%) and TaOE1.1- (18.8%) treated plants (Figure 2A and Figure S2A). Seedlings pre-treated with the TaKO9 presented more damage (28%) when compared to the WT strain-treated seedlings, but did not present the same damage as the *T. atroviride* non-treated plants (Figure 2A and Figure S2A). The TvOE2.2- and TvOE6.2-treated seedlings showed 17.5 and 16.2% of foliar damage, followed by the TvOE2.1- and TvKO2-treated seedlings, which showed 26.2%, and 31% of foliar damage, respectively (Figure 3A and Figure S3A).

When infected with *B. cinerea*, the *T. atroviride* and *T. virens* wild-type strains-treated seedlings showed 26% (Figure 2B and Figure S2B) and 18.6% (Figure 3B and Figure S3B) of foliar damage, respectively. TaOE2.1 conferred high levels (12.8% of foliar damage) of protection against *B. cinerea*, followed by TaOE3.1 (15.5%) and TaOE1.1 (19.6%), respectively (Figure 2B and Figure S2); TvOE2.2 and TvOE6.2 conferred similar protection (~14%), followed by TvOE2.1 (16.6%) (Figure 3B and Figure S3). TaKO9-treated seedlings showed 28% of foliar damage when infected with *B. cinerea* (Figure 2B and Figure S2B), and TvKO2-treated seedlings showed 27.2% of foliar damage (Figure 3B and Figure S3B). However, none of the *T. atroviride* or *T. virens* KO treated plants reached the foliar damage observed in the mock plants (Figures 2B, 3B).

Control plants inoculated with *P. syringae* presented ~28% of leaf damage, whereas root inoculated *T. atroviride* (Figure 2C and Figure S2C) and *T. virens* (Figure 3C and Figure S3C) tomato plants showed reduced foliar damage (20.6 and 24%, respectively). The tomato foliar damage provoked by *Pst* DC3000 was considerably reduced with similar results (10.5%) by TaOE2.1 and TaOE3.1, followed by TaOE1.1 (14.8%) (Figure 2C and Figure S2C). Unexpectedly, the TaKO9 strain-treated plants showed less foliar damage (17%) than those treated with the *T. atroviride* wild-type strain (Figure 2C and Figure S2C). The TvOE6.2-treated seedlings infected with *Pst* DC3000 displayed 14% of foliar damage, followed by TvOE2.1 and TvOE2.2 treated plants with similar results (~18%) (Figure 3C and Figure S3C). Foliar damage of TvKO2-treated seedlings was similar (27.8%) to that of the control seedlings without *Trichoderma*, but inoculated with *Pst* DC3000 (Figure 3C and Figure S3C).

### Sm1 and epl1 differentially modulate defense-related genes in tomato plants

**Figure 4:**
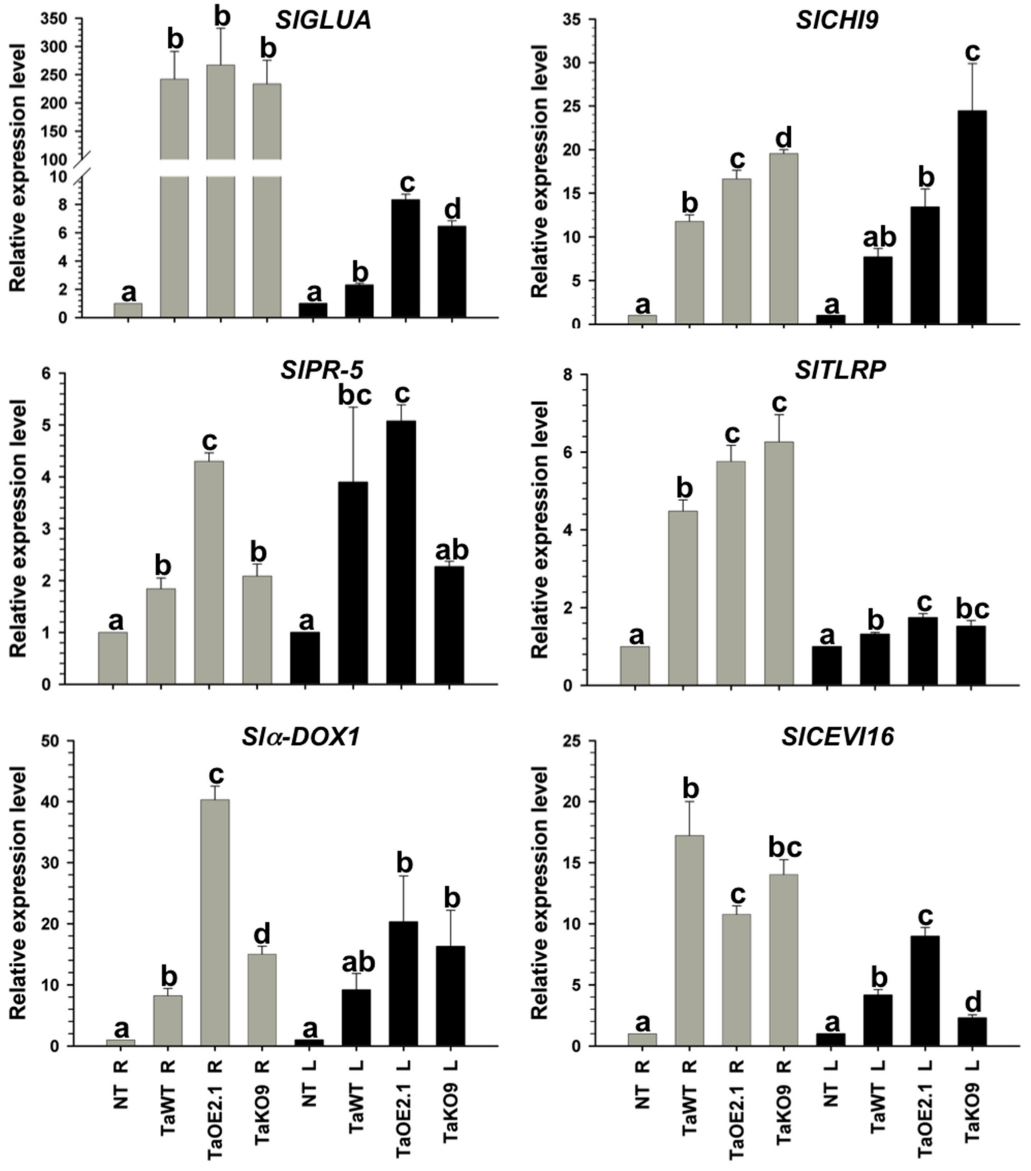
**Epl1 differentially modulates SAR and ISR-related genes in tomato but it is dispensable to induce almost all genes**. Total RNA from roots (R) and leaves (L) of 14-day-old tomato plants inoculated with TaOE2.1 and TaKO9 along with the *T. atroviride* wild-type strain was extracted at 72 h post-inoculation and subjected to RT-qPCR. Non-*Trichoderma* inoculated (NT). Gray bars show relative expression in roots, whereas black bars represent relative expression in leaves. Expression profile of two tomato SAR-related genes (*SlGLUA and SlPR-5*), and four ISR-related genes (*Slα-DOX1, SlCH19, SlTLRP, and SlCEVI16*) was determined. Actin gene *SlTOM51* was used as housekeeping gene. Letter indicates statistically significant differences (analysis of variance, *P* < 0.0001, LSD range test < 0.05).

**Figure 5:**
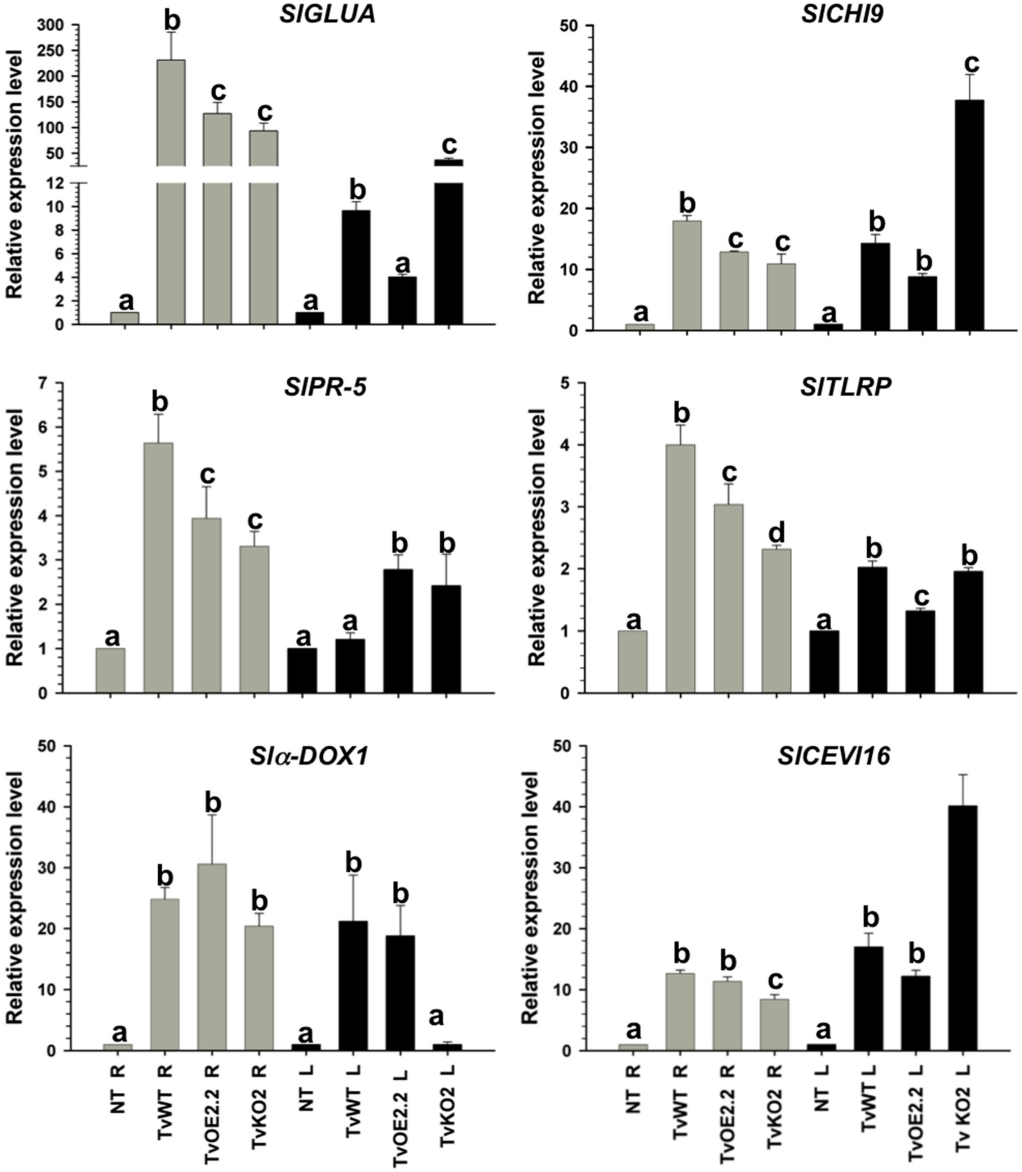
**Sm1 differentially modulates SAR and ISR-related genes in tomato but it is dispensable to induce almost all genes**. Total RNA from roots (R) and leaves (L) of 14-day-old tomato plants inoculated with TvOE2.2 and TaKO2 along with the *T. virens* wild-type strain was extracted at 72 h post-inoculation and subjected to RT-qPCR. Non-*Trichoderma* inoculated (NT). Gray bars show relative expression in roots, whereas black bars represent relative expression in leaves. Expression profile of two tomato SAR-related genes (*SlGLUA and SlPR-5*), and four ISR-related genes (*Slα-DOX1, SlCHI9, SlTLRP, and SlCEVI16*) was determined. Actin gene *SlTOM51* was used as housekeeping gene. Letter indicates statistically significant differences (analysis of variance, *P* < 0.0001, LSD range test < 0.05).

To assign a role to the *sm1* and *epl1* products in the protection of tomato plants against the different pathogens tested, tomato seedlings were root inoculated with the *T. atroviride* wild-type, TaOE2.1, or TaKO9 strain; and the *T. virens* wild-type, TvOE2.2 or TvKO2 strain. Total RNA was extracted from roots and leaves 48, 72, and 96 hpi, and expression of the SAR- and ISR-related genes *SlGLUA* and *SlCHI9*, respectively, were tested by endpoint RT-PCR, detecting maximum expression at 72 hpi with all tested strains (data not shown). Consequently, the 72 hpi point was chosen to further analyze expression of the SAR- (*SlGLUA* and *SlPR5*) and ISR-related genes (*SlCHI9, SlCEVI16*, *Sl*α*-DOX1*, and *SlTLRP*) by RT-qPCR, both, locally (in roots) and systemically (in leaves) (Figures 4, 5).

Almost all selected genes were upregulated in roots of plants treated with all tested strains, whereas in leaves they were induced to a lesser extent (Figures 4, 5). The exception to this transcription pattern was the *SlCHI9* gene in leaves of TaKO9- and TvKO2-treated seedlings, where these strains induced higher transcript levels than those detected for their respective wild-type and OE strains. The *SlCEVI16* gene also showed higher levels of transcript in TvKO2-treated seedlings compared with its corresponding wild-type and OE strains (Figures 4, 5).

The whole set of *T. atroviride* strains induced highest levels of *SlGLUA*, *SlPR-5*, and *Slα-DOX1* in roots and leaves of tomato seedlings compared to the mock case (Figure 4). Although no statistically significant differences were found between *SlGLUA* in TaOE2.1, TaKO9 and wild-type strains-treated plants in roots, and leaves (Figure 4). On the other hand, *SlPR-5*, and *Slα-DOX1* genes showed statistically significant differences in roots and leaves of tomato seedlings treated with the same strains (Figure 4). Roots of *T. atroviride* TaKO9-treated plants showed the highest levels of *SlCHI9* and *SlTLRP* transcription, followed by the TaOE2.1 and wild-type inoculated seedlings. The *SlCHI9* gene displayed a similar behavior of transcription in leaves with all tested strains as compared to its expression levels in roots. However, the *SlTLRP* gene was marginally upregulated in leaves by the TaOE2.1, followed by the TaKO9 and the wild-type treated plants, respectively (Figure 4). The *SlCEVI16* gene showed the highest levels of transcript in roots of *T. atroviride* wild-type strain-inoculated seedlings followed by TaKO9 and TaOE2.1, respectively. Leaves of plants treated with the TaOE2.1 strain showed the highest levels of *SlCEVI16* transcript followed by the wild-type and TaKO9 strains, respectively (Figure 4).

Tomato seedlings treated with the *T. virens* TvKO2 strain showed the lowest levels of transcript for all tested genes in roots, following the induction by the TvOE2.2 and the wild type strains treated plants. The exception to this transcription pattern was the *Slα-DOX1* gene in roots, where no statistically significant differences were found between TvOE2.2, TvKO2 and wild-type strains treated seedlings (Figure 5). Tomato plants treated with the TvOE2.2 strain showed the lowest levels of transcript for *SlGLUA*, *SlCHI9*, *SlTLRP*, and *SlCEVI16* in leaves, sometimes following the induction by either the wild type or the TvKO2 strains treated plants (Figure 5). The *Sl*α*-DOX1* gene, presented the highest levels of transcript in leaves when tomato seedlings were inoculated with the TvOE2.2 and wild type strains, whereas, the TvKO2 was unable to induce *the Slα-DOX1* gene in this tissue (Figure 5). The *SlPR-5* gene showed the highest levels of transcript in leaves of plants treated with both the TvOE2.2 and the TvKO2 strains followed by the wild-type strain (Figure 5).

## Discussion

### *T. virens* and *T. atroviride* promote growth in tomato

The beneficial effects of some *Trichoderma* species on plant growth are well-established (Yedidia et al., 1999; Harman et al., 2004; Shoresh et al., 2005). In this investigation, we showed that the plant beneficial fungi *T. atroviride* (IMI 206040) and *T. virens* (TvG28-9) differentially promote growth of tomato plants. *T. atroviride* promoted a marginally higher plant length than *T. virens*. However, the latter increased significantly more fresh and dry weight than *T. atroviride*. These results indicated that *T. virens* is more effective than *T. atroviride* in promoting biomass gain in tomato plants. Our investigation supports the data reported for the *T. atroviride* and *T. virens* interaction with *A. thaliana*, where both strains induce plant growth (Contreras-Cornejo et al., 2009; Salas-Marina et al., 2011). These direct beneficial effects on plants have also been observed for other *Trichoderma* species interacting with canola, tomato, and maize seedlings (Yedidia et al., 1999; Harman et al., 2004; Shoresh et al., 2005; Tucci et al., 2010). In this context, Tucci et al. (2010) found some differences in plant growth in several tomato lines induced by *T. harzianum* T22 and *T. atroviride* P1, demonstrating that T22 is more effective than P1, and that this feature depends on the tomato genotype. Increasing lines of evidence have shown that the direct effect exerted by *Trichoderma* on plants is through the production of phytohormones, phytohormone-like molecules, volatile organic compounds, secondary metabolites, or by altering the plant phytohormone homeostasis (Gravel et al., 2007; Vinale et al., 2008; Contreras-Cornejo et al., 2009; Viterbo and Horwitz, 2010; Salas-Marina et al., 2011; Olmedo-Monfil and Casas-Flores, 2014; Sáenz-Mata et al., 2014). *Trichoderma* spp. posses several mechanisms to modulate plant growth and development, which, in combination with the plant genotype, could result in different plant phenotypes, indicating that the *Trichoderma* genotype is also important to modulate the plant phenotype.

### Overexpression of epl1 and sm1 in *T. virens* and *T. atroviride*

In this work, we generated KO and OE strains of the *sm1* and *epl1* genes. As expected, those *T. atroviride* and *T. virens* strains with additional copies of the genes showed enhanced levels of transcript as compared to their parental strains, excluding TvOE6.2 whose copy number was 7, whereas the *sm1* transcript levels was 0.5-fold. Intriguingly, as the copy number of *sm1* or *epl1* increased in the genome, the abundance of the corresponding transcript decreased, with the exception of TaOE2.1, which contains five copies of *epl1* and showed higher levels of transcript than TaOE3.1, which contains four copies. Similar results were reported for *T. virens* (Djonovic et al., 2007). In this sense, it has been demonstrated that extra copies of a gene in the genome of filamentous fungi, which induce an overexpression of such gene, could lead to gene silencing (Romano and Macino, 1992). On the other hand, chromatin organization in the integration site and/or chromosomal position can affect the expression of the inserted gene.

### Sm1 and epl1 differentially modulate the induction of systemic disease resistance against different pathogens in tomato plants

Several reports have shown that colonization of plant roots leads to the induction of local and systemic disease resistance against a wide range of phytopathogens (Harman et al., 2004; Shoresh et al., 2010). Accumulating evidences indicate that disease resistance induced by *Trichoderma* spp. is through their MAMPs (Hermosa et al., 2012). Previously, it was reported that Sm1 from *T. virens* induces plant defense response and provides high levels of systemic resistance to cotton plants against the foliar pathogen *Colletotrichum* spp. (Djonovic et al., 2006). Inoculation of maize seedlings with *sm1* OE and KO strains showed that Sm1 is required for induced resistance in this plant (Djonovic et al., 2007). Here we show that *T. atroviride* and *T. virens* induced systemic resistance in tomato against *A. solani*, and *B. cinerea*, and against the hemibiotrophic bacterial pathogen *Pst* DC3000. Both wild-type strains protected tomato seedlings against the three pathogens, whereas *T. atroviride* conferred better protection against *A. solani*, followed by *Pst* DC3000, and *B. cinerea*, respectively, whilst *T. virens* protected tomato seedlings better against *B*. *cinerea*, followed by *Pst* DC3000 and *A. solani*, respectively.

Our results confirm and extend the data of Djonovic et al. (2007), since *sm1* and *epl1* OE induced more efficiently systemic disease resistance against the three tested foliar pathogens, compared to the wild-type strain; whereas the TvKO2 treated seedlings, infected with *Pst* DC3000, were unable of conferring protection to tomato plants. Furthermore, our findings in tomato plants treated with KO strains and infected with *B. cinerea* and *A. solani* clearly indicated that besides Sm1 and Epl1 there are other MAMPs. The above suggests that the *Trichoderma* and the pathogen genotypes are also determinant in the three-way interaction, since almost all treatments of tomato plants with the KO strains conferred less protection than those treated with the WT and OE strains, but never reached the damage observed in the non-treated control. Our results also demonstrated that the Epl1 from *T. atroviride* is able to induce protection against the three tested pathogens. However, it seems that Ep1 plays a minor role as compared to Sm1 from *T. virens*. Interestingly, plants treated with TaKO9 and infected with *Pst* DC3000 showed higher levels of protection than the WT strain. In this regard, other *Trichoderma* MAMPs, including proteins with enzymatic activity (cellulase, xylanase, endopolygalacturonase, an expansin-like protein) and peptaibols, have been reported (Ron et al., 2000; Martinez et al., 2001; Viterbo et al., 2007; Brotman et al., 2008; Morán-Diez et al., 2009). Our results suggest that Epl1 and Sm1 are not the only molecules responsible for the induction of defense responses against these foliar pathogens in tomato, and that there is more than one pathway involved in the plant defense response during the *Trichoderma*-plant-pathogen interaction.

### Sm1 and epl1 differentially modulate defense-related genes in tomato plants

An increase in endogenous SA and the synthesis of *PR* proteins is one of the most common responses triggered in plants following an infection with an inducing microorganism (Van Loon and Van Strien, 1999). The local and systemic disease resistance induced by *T. harzianum* is accompanied by an increase in the enzymatic activity of peroxidase and chitinase, which are involved in the JA/ET and SA response, respectively (Shoresh et al., 2005). In tomato, *SlGLUA*, which encodes for a 35 kDa acidic isoform class III β-1, 3-glucanase, is induced by *B. cinerea* (Benito et al., 1998) as well as by virulent and avirulent races of *Cladosporium fulvum* (van Kan et al., 1992), whereas *SlPR-5*, which encodes for an antifungal osmotin-like protein, is induced by *Fusarium oxysporum* (Rep et al., 2002) and *T. hamatum* 382 (Alfano et al., 2007). These genes are also responsive to the SAR-inducing chemicals SA and INA (2,6-dichloroisonicotinic acid) in tomato (van Kan et al., 1995). Here, we show that *T. atroviride* and *T. virens* were able to successfully induce systemic disease resistance in tomato accompanied by increased expression levels of SA defense-related genes. The expression levels of *SlGLUA* and *SlPR-5* were locally and systemically upregulated when inoculated with all tested strains, although they were induced to a lesser extent in leaves. These results suggest that the products of *SlGLUA* and *SlPR-5* could be involved in the local and systemic disease resistance in tomato mediated by these fungi. Our data suggest also a minor role of Epl1 in the induction of *SlGLUA*, whereas it seems to play a major role on the local and systemic induction of *SlPR-5*. The results of *SlPR*-5 are in agreement with the data reported for the interaction of *T. hamatum* 382 with tomato, in which this microorganism used for biocontrol induced three- to five-fold *SlPR-5* expression in leaves (Alfano et al., 2007). On the other hand, our results indicate that the Sm1 is one of several elicitors of *SlGLUA* and *SlPR-5* since these genes did not show a *sm*1 dose-response behavior in their transcription levels in both, leaves and roots. Contrasting with our data, the inoculation of tomato plants with *Trichoderma harzianum* T-78, did not induce the expression of SA-related gene *SlPR-1a* (Martínez-Medina et al., 2013). We also tested the expression of *SlHMGR* gene that encodes for a 3-hydroxy-3-methylglutaryl CoA reductase gene, whose product is related to the synthesis of sesquiterpene phytoalexin, which was not induced by any of the tested strains. A similar result was found for the SA-related *SlPAL* gene in tomato inoculated with T-78, in spite of an intact pathway for induced resistance is required (Martínez-Medina et al., 2013). Probably, these differences and coincidences depend on both the *Trichoderma* strains, as well as the tomato cultivar.

An alternative pathway in the plant response to pathogen attack is mediated by JA/ET, which is characterized by the production of a cascade of oxidative enzymes (peroxidases, polyphenol oxidases, and lipoxygenases) and the accumulation of low-molecular weight compounds (phytoalexins) (Choudhary et al., 2007). It has been reported that lipoxygenase may generate signal molecules such as JA, methyl-JA, or lipid peroxides, which coordinately amplify specific responses. Furthermore, lipoxygenase activity may also cause irreversible membrane damage, which would lead to the leakage of cellular contents and ultimately result in plant cell death (Croft et al., 1993). Treatment of cotton cotyledons with purified Sm1 resulted in plant autofluorescence and increased levels of phytoalexins as consequence of phenolic compounds oxidation (Hanson and Howell, 2004; Djonovic et al., 2006). The *SlCEVI16* gene encodes for a secreted peroxidase induced by virions and ethephon, where the latter triggers ethylene production in tomato (Gadea et al., 1996). The *Slα–DOX1* gene encodes for an α-dioxygenase, which is involved in the generation of lipid derivates (oxylipins), and is induced by JA and oomycetes (Tirajoh et al., 2004). Our results indicate that Epl1 from *T. atroviride* is more effective locally on the induction of *Slα–DOX1*, but dispensable for its induction, whereas Sm1 from *T. virens* is essential to systemically induce *Slα–DOX1*, but not locally, which indicates that Sm1 could be involved in ISR. Like lipoxygenases, the α-DOX enzymes catalyze the oxygenation of fatty acids to produce oxylipins, including jasmonates, which contribute to basal resistance to bacteria and other pathogens (Hamberg et al., 2005). Interestingly, SA regulates *Slα–DOX1* but not JA in *Arabidopsis*, and whose suppression results in increased bacterial growth (Ponce de Leon et al., 2002), as well as in a diminished SAR response in distal leaves (Vicente et al., 2012). In contrast, the induction of *α–DOX1* in rice plants by *Xanthomonas oryzae* is mediated by JA (Koeduka et al., 2005). It is possible that the systemic induction of *Slα–DOX1* by Sm1 is important to produce oxylipins to counteract *B. cinerea* and *Pst* DC3000 by means of SA and/or JA pathways. In this regard, the simultaneous induction of these signal transduction pathways by *T. atroviride* and its effectiveness to counteract these pathogens has been reported previously (Salas-Marina et al., 2011).

*SlCEVI16* was induced locally by both sets of WT, OE, and KO strains, without a correlation regarding its Epl1 or Sm1 copy number, whereas in leaves of plants treated with *T. atroviride* strains, the induction of *SlCEVI16* agreed with the *sm1* copy number. This result indicates that the Epl1 could play a major role on the systemic induction, but not locally in *SlCEVI16* induction during *T. atroviride*-tomato interaction, and this in turn could be important to counteract soil and airborne pathogens, including *B. cinerea* and *Pst* DC3000. On the contrary, plants treated with the different *T. virens* strains did not show a dose-response behavior in the transcription levels of *SlCEVI16* in leaves, indicating a minor role of Sm1 on the induction of this gene which could be dispensable to attack *A. solani* and *B. cinerea*. It has been demonstrated that plant peroxidases are involved in the response to pathogens mediated by JA/ET, whose activity has been related to resistance responses, including lignifications and suberization, cross-linking of cell wall proteins, generation of reactive oxygen species, and phytoalexins synthesis, the latter show antifungal activity themselves (Bolwell and Wojtaszek, 1997; Quiroga et al., 2000; Caruso et al., 2001).

The *SlCHI9* and *SlTLRP* genes showed local and systemic induction when inoculated with all the *Trichoderma* strains, but did not show a correlation with the *sm1* or *epl1* copy number. TaOE2.1 induced marginally higher levels of *SlCHI9* and *SlTLRP* both, locally and systemically compared to the wild-type, whereas the overexpression of *sm1* leads to a negative effect on the expression of such genes. These results indicate that Sm1 does not play an important role in the induction of these genes, whilst Epl1 seems to play a role on the induction of ISR-related genes. Intriguingly, in almost all cases TvKO9 induced high levels of *SlCHI9* and *SlTLRP* locally and systemically, whereas *SlCHI9* showed high transcript levels in leaves, but not the *SlTLRP* when inoculated with the TvKO2. In this regard, Djonovic et al. (2007) found that SA-related genes, *PR-1* and *PR-5*, as well as *AOS* and *OPR7*, which are related to JA in maize, were downregulated by OE strains as compared to the wild-type after challenging maize plants with *C. graminicola*. Interestingly, in some cases the expression levels of such genes were lower than in plants treated with the KO strain.

Our results showed that the expression levels of *GLUA* were higher in roots than in leaves. However, the rest of the genes selected showed, in general, marginally higher level of expression in roots than in leaves. Similar results have been obtained in similar studies using other plant species inoculated with *Trichoderma* spp. (Yedidia et al., 2003; Shoresh et al., 2005, 2006; Salas-Marina et al., 2011). It has also been shown that *Trichoderma* treated plants, when inoculated with an airborne pathogen respond very strongly, displaying much higher levels of expression of defense related genes (Viterbo et al., 2007; Mathys et al., 2012; Perazzolli et al., 2012). We hypothesized, that upon inoculation of tomato plants treated with the different *Trichoderma* strains with an airborne pathogen, the induction of such genes would be higher than in seedlings inoculated only with *Trichoderma*, due to the priming effect of *Trichoderma*.

Based on the systemic protection experiments, our study provides genetic evidence that the elicitors of defense response, Epl1 and Sm1, are able to induce protection against hemibiotrophic and necrotrophic pathogens, which means that they are inducing ISR and SAR. The molecular analysis of SAR- and ISR-related genes led us to conclude that the Sm1 and Epl1 proteins play a minor role in the induction of basal levels of these genes, since both *T. atroviride* and *T. virens* KO strains were able to induce five of six genes in tomato, with the exception of *SlCEVI16* by TaKO9 and *Slα–DOX1* by TvKO2, whose transcript levels were similar to those detected in mock plants. Taking into account that *SlCEVI16* and *Slα–DOX1* genes are induced by the same signal transduction pathway, indicates that each, Sm1 and Epl1, has different specific targets in the same pathway to counteract pathogens with different life style. Therefore, our work indicates that the induction of ISR by *T. virens* and *T. atroviride* could be more important than the SAR signal to counteract pathogens as demonstrated by Martínez-Medina et al. (2013). More studies need to be undertaken to clarify this proposal.

### Conflict of interest statement

The authors declare that the research was conducted in the absence of any commercial or financial relationships that could be construed as a potential conflict of interest.
