# Supplementary figures and images for: The Epl1 and Sm1 proteins from Trichoderma atroviride and Trichoderma virens differentially modulate systemic disease resistance against different life style pathogens in Solanum lycopersicum
Source: Front Plant Sci. 2015 Feb 23;6:77. doi: 10.3389/fpls.2015.00077 (PMC4337343; doi:10.3389/fpls.2015.00077)

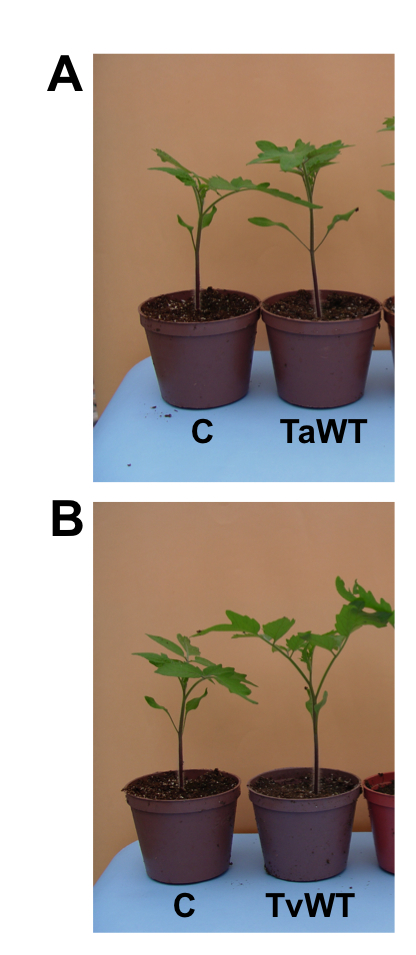

Supplement: Supplementary file 1 [file Image1.JPEG]

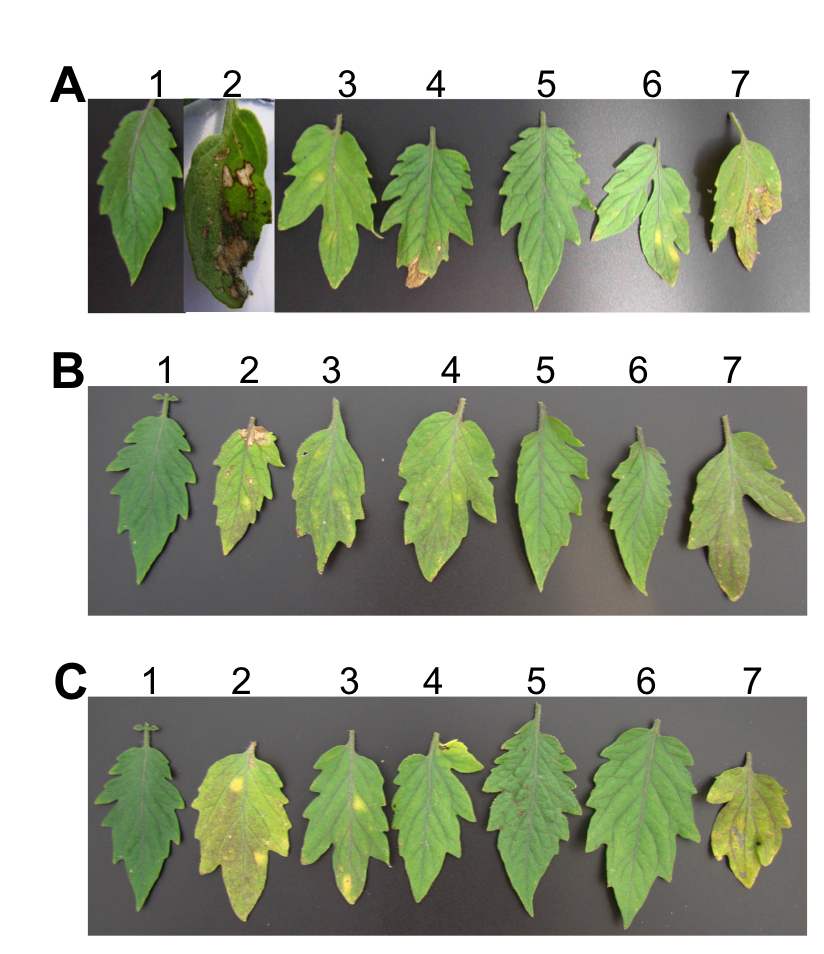

Supplement: Supplementary file 2 [file Image2.JPEG]

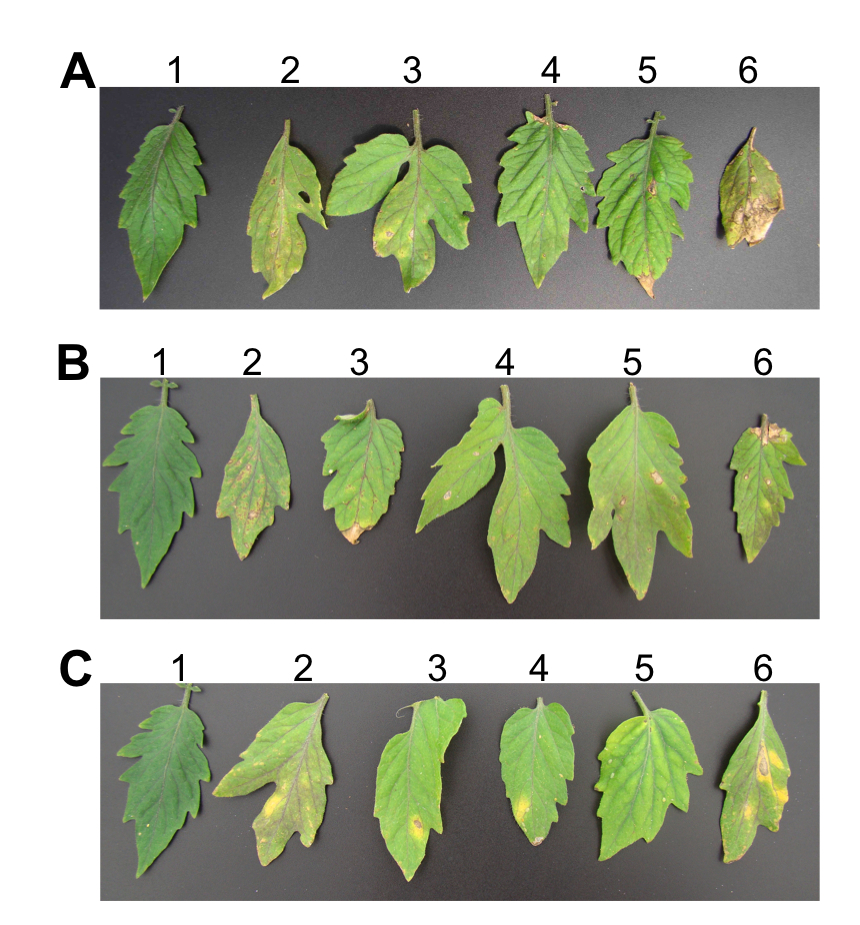

Supplement: Supplementary file 3 [file Image3.JPEG]
